# Dual Monitoring of Blood Acetylcholinesterase Content and Catalytic Activity Utilizing Fluorometry-Integrated Surface Plasmon Resonance

**DOI:** 10.3390/bios15020118

**Published:** 2025-02-17

**Authors:** Yuanyuan Xie, Yifei Hou, Mengwei Hu, Hongzhuan Chen, Hao Wang, Lanxue Zhao, Jianrong Xu

**Affiliations:** 1 School of Integrative Medicine, Shanghai University of Traditional Chinese Medicine, Shanghai 201203, China; 2 Institute of Interdisciplinary Integrative Medicine Research, Shanghai University of Traditional Chinese Medicine, Shanghai 201203, China; 12022193@shutcm.edu.cn; 3 Department of Pharmacology and Chemical Biology, Shanghai Jiao Tong University School of Medicine, Shanghai 200025, China; 4 Clinical Trial Center of Zhongnan Hospital, Wuhan University, Wuhan 430071, China; 5 Shuguang Lab of Future Health, Shuguang Hospital, Shanghai University of Traditional Chinese Medicine, Shanghai 201203, China; 6 Shanghai Frontiers Science Center of TCM Chemical Biology, Shanghai University of Traditional Chinese Medicine, Shanghai 201203, China

**Keywords:** surface plasmon resonance, Alzheimer’s disease, cholinesterase inhibitor, safety monitoring

## Abstract

Acetylcholinesterase inhibitors (AChEIs), particularly donepezil, are commonly used to treat mild-to-moderate Alzheimer’s disease (AD). However, drug accumulation during long-term use could change AChE activity and content, leading to peripheral side effects and prompting medication discontinuation. However, there are a lack of methods to simultaneously determine the content and catalytic activity of AChE. By using phosphatidylinositol-specific phospholipase C to strip AChE from erythrocyte surfaces, we developed a novel method combining surface plasmon resonance and fluorescence detection for the simultaneous detection of AChE content and activity, producing stable, reliable, and accurate results. The established determination range spans from 263.37 ng/mL to 3000 ng/mL (4.05 nM to 46.15 nM) for concentration, and from 39.02 mU/mL to 1000 mU/mL for activity. Compared to traditional methods, this approach simplifies operations, reduces detection time, expands the dynamic range, and lowers detection limits, potentially advancing AChE-related research and supporting clinical diagnostics and drug development.

## 1. Introduction

Acetylcholine (ACh) plays a major role as a neurotransmitter and neuromodulator throughout the central nervous system (CNS), as well as in the peripheral system, and is involved in a wide range of body functions, such as cardiac homeostasis, gland secretion, and nervous system functions [1,2,3]. Acetylcholinesterase (AChE) hydrolyzes ACh into choline and acetate, thereby terminating neurotransmission and preventing continuous nerve impulses at nerve terminals, making it a prime therapeutic target for diseases that occur with a cholinergic deficit, prominently Alzheimer’s disease (AD) and myasthenia gravis (MG) [4,5].

Acetylcholinesterase inhibitors (AChEIs) such as donepezil, rivastigmine, and galantamine are first-line therapeutic agents [6,7,8]. However, when AChEIs accumulate peripherally, they could inhibit the activity of AChE in the blood, leading to a series of adverse reactions, including gastrointestinal disturbances [9], bronchospasm, rapid breathing, and the exacerbation or induction of asthma and urinary tract infections. In severe cases, bradycardia, cardiac conduction block, and QT interval prolongation may occur [10,11,12,13], resulting in serious consequences [14]. Moreover, AChE levels and activity in the blood have long been regarded as a clinical indicator of recovery from organophosphorus pesticide poisoning [15]. AChE in erythrocytes is primarily located on the cell membrane, where it plays a critical role in hydrolyzing acetylcholine, thereby maintaining neurotransmitter balance and supporting erythrocyte function [16]. Although other cells in the plasma, such as lymphocytes, and certain nervous system cells, such as neurons, may contribute a small amount of AChE, erythrocytes remain the most significant source of total blood AChE activity [17,18,19,20]. The expected level of AChE in blood varies depending on the population and measurement methods, but generally falls within the range of 2 to 5 U/mL for activity in healthy individuals [21,22,23,24]. These levels can be influenced by various factors, including age, sex, and health conditions, such as neurodegenerative diseases or exposure to organophosphorus compounds, which can inhibit AChE activity [18]. Understanding the distribution and baseline levels of AChE in blood is crucial for interpreting changes induced by inhibitors or pathological conditions. Currently, there is no established clinical method for the rapid early warning and monitoring of AChE content and activity in the blood.

The determination of AChE content has mainly, and primarily, relied on the Western blot and enzyme-linked immunosorbent assay (ELISA), and the assessment of activity has utilized Ellman’s assay, fluorescence analysis, and thin-layer chromatography (TLC) [25,26]. However, these approaches are all time-consuming and manpower-demanding. Moreover, the determination of content and activity cannot be obtained simultaneously, which fails to meet the requirements for rapid and combined measurement in acute situations like organophosphorus pesticide poisoning. Additionally, the reproducibility of the results is highly dependent on the operator. Therefore, it is necessary to establish a sample-sensitive and reliable method for the simultaneous determination of AChE content and activity.

Surface plasmon resonance (SPR) is an optical, label-free technique for the real-time monitoring of biomolecular interactions. In SPR experiments, a ligand is typically immobilized on the surface of a sensor chip coated with a thin metal film, and a solution containing analyte is then flowed over the chip surface. When binding or dissociation occurs between the two molecules, changes in the mass of biomolecules bound to the surface result in proportional changes in the refractive index and a shift in the SPR angle. The detection of this shift allows for the acquisition of the kinetic, affinity, and thermodynamic parameters of the molecular interactions [27,28]. SPR, characterized by its label-free nature, real-time monitoring capability, and high sensitivity, has been widely applied in biochemistry, drug development, and medical diagnostics. For instance, SPR has been used to measure the concentration of CXCL12 in human urine samples [29] and determine the affinities and kinetic rates of antibodies binding to different low-abundant native biomarkers like NFL in CSF, GDF15 in serum, or sTREM-1 in plasma samples [30]. SPR stands out as a versatile tool for probing biomolecular interactions. Its real-time, label-free, and highly sensitive detection capabilities have led to significant advancements in various fields. Its applications extend into biomedical research, environmental monitoring, food safety testing, disease diagnosis, nanotechnology, materials science, and even cell biology, highlighting its broad utility and critical importance across diverse domains [31,32,33,34].

In this study, we developed a novel method for the simultaneous detection of AChE content and activity by integrating SPR with a fluorescence detector. The SPR chip captures AChE with high sensitivity and specificity, providing a robust platform for quantification. The hydrolysis product then reacts with a chromogenic reagent to generate a fluorescence signal, quantitatively measured by a fluorescence detector. This approach allows us to obtain both concentration and activity information of AChE in a single workflow.

Compared to traditional methods such as ELISA and Ellman’s assay, our new approach significantly simplifies the operational procedure, reduces detection time, and offers enhanced sensitivity and specificity. Furthermore, the integration of SPR endows our detection method with a wider dynamic range and lower detection limit. Correlating AChE quantity with its enzymatic activity holds promise for clinical diagnostics, particularly in the early detection of diseases associated with AChE dysfunction, and supports the development of targeted therapeutics.

## 2. Materials and Methods

### 2.1. Materials and Apparatus

The materials used in this study were as follows: phosphate-buffer solution (PBS) (Double-Helix, Shanghai, China), TWEEN^®^ 20 (Sigma-Aldrich, St. Louis, MO, USA), acetylthiocholine (ATCI) (Sigma-Aldrich, St. Louis, MO, USA), 5,5-Dithiobis (2-nitrobenzoicacid) (DTNB) (Sigma-Aldrich, St. Louis, MO, USA), human AChE standard (R&D systems, Minneapolis, MN, USA), N-hydroxysuccinimide (NHS) (Cytiva, Uppsala, Sweden), N-(3-dimethylaminopropyl)-N′-ethylcarbodiimide hydro-chloride (EDC) (Cytiva, Uppsala, Sweden), acetate buffer pH 5.5 (Cytiva, Uppsala, Sweden), ethanolamine (Cytiva, Uppsala, Sweden), mouse monoclonal anti-AChE antibody MAB303 (Sigma-Aldrich, St. Louis, MO, USA), mouse monoclonal anti-AChE antibody A11 (Santa Cruz, Dallas, TX, USA), rabbit polyclonal anti-AChE antibody RP01 (Sino Biological, Beijing, China), mouse monoclonal anti-AChE antibody 4E11 (Novus Biologicals, Littleton, CO, USA).

SPR assays were operated on a CM5 SPR chip incorporated in a Biacore T200 SPR instrument (Cytiva, Uppsala, Sweden), A multi-functional enzyme-labeling instrument (Thermo Fisher, Waltham, MA, USA) was used to measure the fluorescence intensity of the reaction solution.

All solutions were prepared with ultrapure water obtained from a Laboratory ultrapure water system (Millipore, Billerica, MA, USA) and filtered using 0.22 µm membrane filter before use. All experiments carried out with Biacore T200 were controlled at 25 ± 1 °C.

### 2.2. Human Blood

All procedures performed in this study were in accordance with the ethical standards of the national committee and with the 1964 Declaration of Helsinki. The human blood samples used in this study (*n* = 15) were obtained from different donors and provided by the Regional Blood Donation Center.

### 2.3. Immobilization of Anti-AChE Antibody on the CM5 Chip

The anti-AChE antibody was immobilized on the surface of CM5 chip channel Fc2, and channel Fc1 was used as the reference channel. A mixture of 400 mM EDC/100 mM NHS (1:1, *v*/*v*) were injected for 7 min at a flow rate of 10 μL/min to activate the chip surface, making the chip surface carry a negative charge. The anti-AChE antibody was diluted to a concentration of 50 µg/mL in pH 5.5 acetate buffer, and then injected for 7 min at 10 μL/min to bind with the carboxyl group on the chip surface. 1 M ethanolamine-HCl pH 8.5 was injected at a flow rate of 10 μL/min for 7 min to block the remaining activated groups and prevent nonspecific binding.

### 2.4. Screening of Anti-AChE Antibody

Four commercially available AChE antibodies (approximately 150 kDa) were acquired and immobilized on the CM5 chip individually. Subsequently, 1 μg/mL AChE (65 kDa) standard was used to flow through the chip surface at a flow rate of 10 μL/min. After normalization with the reference channel, any antibody showing a pattern of rapid binding followed by quick dissociation, or displaying unstable binding characteristics with excessively fast dissociation rates, would be identified as unsuitable for the assay method under evaluation. Antibodies that maintain stable interactions with AChE and exhibit minimal dissociation from the chip surface are deemed appropriate for advancing to subsequent experimental stages, thereby qualifying as suitable candidates for this detection method.

### 2.5. Establishment of Standard Curve of AChE Gradient Concentration

The AChE standard was diluted to 3 μg/mL, then 7 gradient concentrations were diluted with PBST (10 mM PBS, 0.05% Tween 20, pH 7.4) buffer to an increase of 1.5 times. Samples were injected from low to high concentration at the flow rate of 10 μL/min. During sample analysis, the association time was set up to 120 s, and the dissociation time to 240 s. Regeneration was performed using a glycine hydrochloride buffer (Gly-HCl), with a pH 2.1 for 30 s. After subtracting the response value at zero concentration, the content standard curve with the response values on the y-axis and the concentrations on the x-axis was plotted and fitted by four-parameter fitting.

### 2.6. Establishment of Standard Curve of AChE Gradient Activity

The AChE standard was diluted to 1 U/mL, then 9 gradient concentrations were diluted with PBST buffer at to an increase of 1.5 times. Samples were injected from low to high concentration at the flow rate of 10 μL/min. During sample analysis, the association time was set to 120 s, and the dissociation time to 240 s. Under the “inject and recover” mode, 1 mM ATCI (289.18 g/mol) was injected for recovery, incubated for 60 s, stabled for 60 s, and the recovery process was cycled for 10 times. The reaction time of AChE with ATCI should be as long as possible to obtain stronger fluorescence signal. After collecting the reaction product, the thiol green indicator was added, and the fluorescence intensity was measured. After the recovery, 10 mM glycine hydrochloride buffer (Gly-HCl), pH 2.1, was injected for 30 s for regeneration, and the next sample was detected. The fluorescence intensity of reaction solution was measured for 30 min continuously to observe whether the fluorescence intensity of the reaction liquid was stable. The sample activity was plotted on the x-axis, with fluorescence intensity on the y-axis, yielding the activity standard curve. The curve was fitted by four-parameter fitting.

### 2.7. The Expression and Purification of Phosphatidylinositol-Specific Phospholipase C (PIPLC)

Based on the gene sequence of PIPLC from *Bacillus cereus* [35,36] in the NCBI database, codon optimization was performed according to the codon preference of *Escherichia coli*. The corresponding gene sequence was synthesized and a gene expression vector pET-22b (+) was constructed. The recombinant plasmid was transformed into the recipient strain *E. coli* BL21 (DE3), and the target gene PIPLC was induced by adding isopropyl thio-β-D-galactoside (IPTG). After detection, it was found that the PIPLC fusion protein containing the His tag existed as a soluble protein in the supernatant of bacterial cell disruption, which was consistent with expectations. After preliminary optimization of the induction conditions, the optimal induction conditions were found to be as follows: inoculation at 5% volume fraction, waiting for the bacteria to grow until the OD600 reached 0.5, and induction at 16 °C with 0.3 mmol/L IPTG for 24 h. The protein was purified using the His tag. The enzyme activity is measured using phosphatidylinositol as the substrate and the purified PIPLC had an enzyme activity of 500 U/mg.

### 2.8. Pretreatment of Blood Samples

Because AChE is bound to the erythrocyte membrane through phosphatidylinositol in blood, a certain pretreatment is needed to dissociate it from the erythrocyte membrane. Phosphatidylinositol can be degraded by PIPLC, and thereby AChE can be dissociated from the erythrocyte membrane [37]. In this study, whole blood was first centrifuged at 4 °C at 4000 rpm for 5 min to separate the plasma from erythrocytes, with the supernatant (plasma) discarded. Physiological saline, in a volume three times that of the erythrocytes, was then added to the pelleted erythrocytes, and the mixture was centrifuged again at 4000 rpm for 5 min. This washing step was repeated three times to ensure the thorough removal of any residual plasma components. After the final wash, the erythrocytes were resuspended in an appropriate volume of physiological saline, and PIPLC was added to degrade phosphatidylinositol, facilitating the dissociation of AChE from the erythrocyte membrane. Following incubation with PIPLC, the mixture was centrifuged one last time to pellet any remaining intact erythrocytes or debris. The supernatant, containing the dissociated AChE, was carefully collected and stored at 4 °C until further use.

#### 2.8.1. Optimal Concentration for PIPLC Incubation

PIPLC was diluted to a final concentration of 0.1–100 μg/mL using PBS. After washing, 50 μL of erythrocytes were mixed with 150 μL of the PIPLC gradient solution, gently agitated, and incubated at 37 °C for 30 min. The mixture was then centrifuged at 4000 rpm for 5 min, and the supernatant was collected. The negative control consisted of the supernatant from the same volume of blank PBS mixed with erythrocytes, while the positive control was a 1 U/mL AChE standard. The activity of the erythrocyte supernatant incubated with gradient concentrations of PIPLC was determined using the Ellman’s method.

#### 2.8.2. Optimal Volume for PIPLC Incubation

We took 50 μL of washed erythrocytes and mixed them with 150, 250, 350, and 450 μL of PIPLC (final concentration 1 μg/mL), gently reversed and mixed well, incubated for 10 min at 37 °C, then centrifuged at 4000 rpm for 5 min at 4 °C, and the supernatant was determined. The negative control was the supernatant from the same volume of blank PBS buffer mixed with erythrocytes, and the positive control was a 1 U/mL AChE standard. The activity of the erythrocyte supernatant incubated with different volume ratios of PIPLC was determined by Ellman’s method. Because the volume is different, the final result was counted by activity.

#### 2.8.3. Optimal Time for PIPLC Incubation

We took 50 μL of washed erythrocytes and mixed them with 150 μL PIPLC (final concentration 1 μg/mL), gently reversed and mixed well. Incubated for 0, 1, 2, 5, 10, 15, and 30 min at 37 °C, centrifuged at 4000 rpm for 5 min at 4 °C and the supernatant was collected. The negative control was the supernatant of the same volume of blank PBS buffer mixed with erythrocytes, and the positive control was 1 U/mL AChE standard. The activity of the supernatant of erythrocytes incubated with PIPLC at different times was determined by Ellman’s method.

#### 2.8.4. Routine Analysis of the Activity of AChE in Erythrocyte Membrane

We took 50 μL of the washed erythrocytes and extracted the erythrocyte membrane using ultrasonic fragmentation. We discarded the supernatant and diluted the remaining part with PBS to a final volume of 200 μL. Then, we took another 50 μL of washed erythrocytes and mixed them with 150 μL of PBS or PIPLC (final concentration 1 μg/mL). We incubated them at 37 °C for 10 min. Subsequently, we centrifuged them at 4000 rpm for 5 min to obtain the supernatant. In order to eliminate false positives, 150 μL PIPLC (final concentration 1 μg/mL) was mixed with 50 μL physiological saline and treated in the same way. Finally, we determined the activity of AChE by Ellman’s method.

### 2.9. Methodology Validation

In the sample mode, low- (400 ng/mL, 60 mU/mL), medium- (800 ng/mL, 320 mU/mL), and high- (2000 ng/mL, 800 mU/mL) concentration AChE standards were chosen, and each concentration was divided into five parallel samples. The flow rate was 10 μL/min, the binding time was 120 s, the dissociation time was 300 s, and the response value was the output. In the inject and recovery mode, the 1 mM ATCI was injected for recovery, incubated for 60 s, stabilized for 60 s, and the recovery process was cycled 10 times. The reaction solution, with thiol green indicator added, was introduced to the fluorescence detector to determine the fluorescence intensity of the reaction solution. The response value and fluorescence intensity were substituted into the standard curve of content and activity to obtain the intra-day difference. The method was repeated for three days and the inter-day difference was obtained.

The AChE standard was used to prepare the minimum limit of quantitation concentration. Five samples were paralleled. The intra-day difference and inter-day difference in the lowest limit of quantity were determined by the above methods.

### 2.10. Enzyme-Linked Immunosorbent Assay (ELISA)

AChE concentration in human blood was analyzed using an acetylcholinesterase concentration ELISA kit (Jiancheng, Nanjing, China) according to the manufacturer’s instructions.

### 2.11. Ellman’s Assay

Ellman’s assay is a commonly used method for measuring the activity of AChE. This method quantitatively analyzes enzyme activity by measuring the yellow product formed from the reaction of thiocholine, generated by the hydrolysis of acetylthiocholine by AChE, with DTNB. Briefly, we prepared a series concentrations of AChE standards by PBS buffer. Then, we added an equal volume of DTNB solution to each sample and mixed well. We incubated them at room temperature for 5 min to allow the reaction to proceed. We measured the absorbance at 490/520 nm using a spectrophotometer. We recorded the absorbance values of each concentration and plotted a standard curve. Finally, for the test samples, we repeated the above steps and calculated AChE activity based on the standard curve.

### 2.12. Sample Determination

The whole blood samples of different individuals were incubated with donepezil (final concentration 0, 15.63, 31.25, 62.5, 125, 250, 500 ng/mL) or DDPV (final concentration 0, 100 nM, 1 μM, 10 μM, 100 μM, 1 mM) at 37 °C. The remaining processing steps were the same as before. The response value after 120 s of sample injection was substituted into the content standard curve, and the stable fluorescence intensity value was substituted into the activity standard curve to obtain the content and activity value of the sample, respectively.

## 3. Results and Discussion

### 3.1. The Assay Strategy for Simultaneously Measuring AChE Content and Activity with SPR

**Figure 1:**
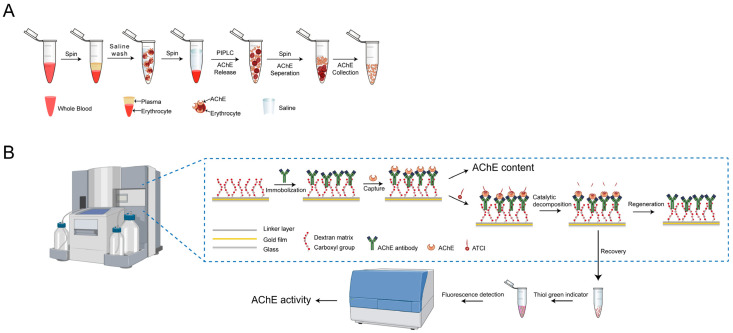
Schematic diagrams. (**A**) Diagram of human blood samples preprocess. (**B**) Schematic of SPR integrated with a fluorescence detector detection for AChE content and activity.

In this study, we developed an integrated approach for detecting both the concentration and activity of AChE. SPR was used to measure the AChE concentration by immobilizing the anti-AChE antibody on a sensor chip, with the binding amount directly correlating to the AChE concentration. A standard curve was established by plotting known concentrations of AChE against the corresponding SPR response values. Concurrently, the fluorescence detection was employed to measure AChE activity. Since the SPR instrument (Biacore T200) alone is not capable of fluorescence detection, we chose to use a multifunctional microplate reader to simultaneously measure AChE activity in our experiments. In future applications, it is feasible to directly integrate a fluorescence detector into the flow path of an SPR instrument to enable the faster combined detection of AChE concentration and activity. Therefore, by integrating SPR with a fluorescence detector (Figure 1), both AChE activity and content could be obtained rapidly and simultaneously through a streamlined testing process.

The main process of the SPR-based AChE detection technique was as follows: firstly, the anti-AChE antibody was immobilized to the sensor chip, and the blood sample was analyzed after pretreatment. The antibody immobilized on the chip could specifically capture AChE. According to the binding amount, the content of AChE in blood could be obtained. The substrate ATCI was then injected and hydrolyzed by AChE. The hydrolyzed product of ATCI, thiocholine, was recycled and mixed with a chromogenic agent, a thiol green indicator. The reaction product, with thiol green indicator added, was then introduced into the fluorescence detector, where the fluorescence intensity was measured to determine the AChE activity.

Although the two measurements were performed on different instruments, our method was designed to simultaneously assess both the concentration and activity of AChE within a single experimental framework. Compared to separately using ELISA and Ellman’s assay to measure AChE concentration and activity, our method offers key advantages: it simultaneously assesses both parameters within a single experimental framework, reducing sample consumption, minimizing operational variability, and improving data consistency.

### 3.2. Immobilization of Anti-AChE Antibody

In this study, the CM5 chip was used to immobilize the anti-AChE antibody, which could specifically recognize and capture AChE molecules in the blood sample. The CM5 chip is a commonly used SPR sensor chip with a carboxymethylated dextran matrix on its surface. This matrix allows for the covalent immobilization of various biomolecules, such as antibodies, proteins, or other macromolecules. The coupling of anti-AChE antibody was carried out at pH 5.5 and 50 μg/mL. As shown in Figure S1, the coupling amount of the anti-AChE antibody was about 6000 RU, meeting the requirements of subsequent determination. In SPR experiments, the response unit (RU) is a measure of changes in light reflection, indicating molecular interactions occurring on the sensor surface. Typically, one RU corresponds to an increase of 1 pg/mm^2^ in the biomolecular mass on the sensor surface. The change in RU is proportional to the molecular mass and the amount of bound analyte, making it a critical parameter for assessing the binding interactions between analytes and ligands, as well as for analyzing kinetic parameters such as the association rate constant (ka) and dissociation rate constant (kd).

### 3.3. The Binding and Regeneration of AChE to Anti-AChE Antibody

**Figure 2:**
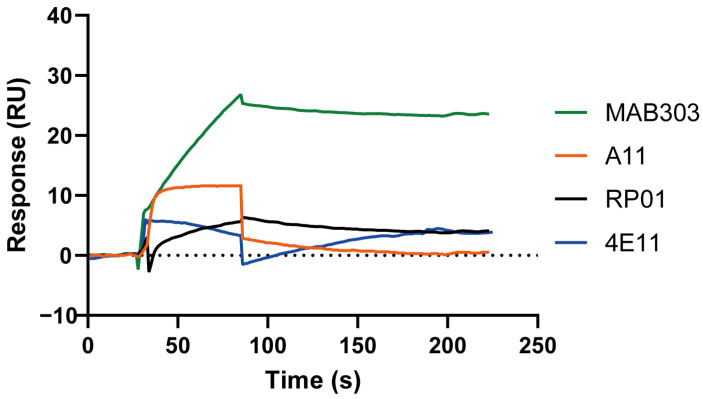
The binding of AChE to different anti-AChE antibodies immobilized on the CM5 chip.

As shown in Figure S2, AChE was injected at a flow rate of 10 μL/min and bound to the antibody. After binding, the signal of AChE was very stable without an obvious dissociation process, which ensured that AChE would be sufficiently stable when ATCI subsequently injected. Four AChE antibodies were screened and MAB303 was identified as the most stable antibody for AChE capture, which was used in subsequent experiments (Figure 2). This is because it exhibited significant binding to AChE and demonstrated slow and stable dissociation, indicating that the MAB303 antibody could effectively recognize and capture AChE. When injecting PBS or BSA, no significant binding was observed; however, a significant binding signal emerged upon injecting the AChE standard and remained stable after sample injection. This indicated that the anti-AChE antibody MAB303 could specifically capture AChE. Furthermore, when injecting erythrocytes treated with PIPLC, a significant binding signal was also observed, and it remained stable after sample injection (Figure S3). The regeneration process was crucial for the determination of AChE. By dissociating AChE from the immobilized antibodies, the chip could be reused for repeated detection of blood samples with different concentrations and from different individuals, significantly enhancing the efficiency and cost-effectiveness of the assay. Generally, regeneration could be carried out in acidic, alkaline, or neutral high-concentration salt buffers. The regeneration condition depended on the nature of the immobilized protein and the ligand. If regeneration was incomplete and the binding sites were occupied, it would reduce the output response value; if regeneration conditions were not appropriate and damaged the activity of the antibody, it would further affect the determination of subsequent samples. Gly-HCl, pH 2.1, was optimized as the regeneration condition in this study. After each sample was determined, Gly-HCl, pH 2.1, was injected at a flow rate of 10 μL/min for 30 s, and AChE was completely dissociated from the antibody without affecting its activity.

### 3.4. Establishment of a Standard Curve for AChE Content

**Figure 3:**
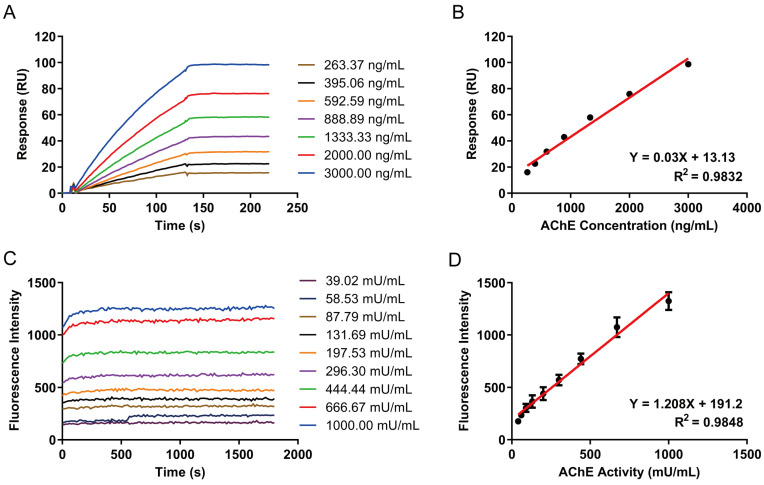
The standard curve of AChE concentration and activity. (**A**) The binding curves of AChE at gradient concentrations of the anti-AChE antibody by SPR. (**B**) The calibration curve of AChE at gradient concentrations. (**C**) The fluorescence intensity of AChE at gradient activity. (**D**) The calibration curve of AChE at gradient activity.

The binding of different concentrations of AChE standards and anti-AChE antibody could output different binding signals. When the binding was completed, the bound AChE was very stable in the dissociation time, and the curve was plateau-shaped (Figure 3A). Therefore, when the combination was completed, the standard curve of AChE content was established by taking the response value of the plateau phase as the y-axis and the concentration as the x-axis (Figure 3B), which enabled the calculation of AChE concentrations in subsequently tested human blood samples. As shown in Figure 3B, the binding curves of samples from the lowest to the highest concentration were linear during the 120 s binding process, which indicated that even the highest concentration was far from saturation, and the response value was linearly correlated with the concentration. Since the blank itself had a trace response, it was necessary to subtract the response value of the blank throughout the whole process, which was also helpful to eliminate the system error of the instrument.

Due to the optimization of regeneration conditions, AChE could be completely removed without affecting antibody activity. Therefore, in clinical applications, once the antibodies are immobilized on the chip, a single calibration curve can be established and reused to calculate the concentrations of multiple blood samples. However, with increasing usage, the activity of the antibodies and the chip may gradually and slightly decline. Therefore, it is necessary to monitor and record their performance during use to ensure timely replacement of the chip and re-immobilization of antibodies.

### 3.5. Establishment of a Standard Curve for AChE Activity

When AChE with varied activity were captured by AChE antibodies on the chip surface, the ATCI was injected at a flow rate of 10 μL/min. ATCI was hydrolyzed to acetic acid and thiocholine by AChE. Thiocholine was then reacted with the thiol green indicator, and a strong fluorescence could be detected at excitation and emission wavelength of 490 nm and 520 nm, respectively. Therefore, the reaction solution can be introduced to a fluorescence detector to measure the fluorescence intensity. The sensitivity of this method was limited because the reaction time of ATCI in the instrument was short and the amount of AChE was low, which led to constrained reaction products. In order to increase the amount of reaction products, thereby improving the sensitivity of the method, the catalytic reaction process was cycled 10 times, and all products were pooled with the thiol green indicator together and introduced to the fluorescence detector. The fluorescence intensity of the reaction solution was continuously measured in the fluorescence detector for 30 min. It can be observed that the fluorescence intensity output by AChE samples with different activities followed a similar gradient and were linearly correlated with the activity (Figure 3C). The fluorescence intensity was stable within 30 min. Therefore, the standard curve of AChE activity could be obtained by using the fluorescence intensity of minus zero concentration at 30 min (1800 s) as the y-axis and activity as the x-axis (Figure 3D).

### 3.6. Pretreatment Conditions of Blood Samples

**Figure 4:**
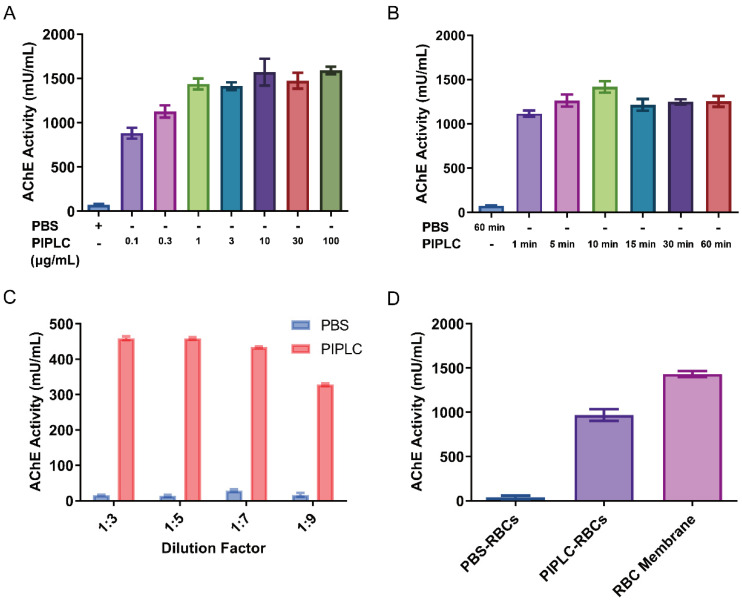
Optimization of pretreatment of blood samples. (**A**) The activity of AChE on erythrocytes at different PIPLC incubating time. (**B**) The activity of AChE on erythrocytes at different PIPLC incubating concentration. (**C**) The activity of AChE on erythrocytes at different PIPLC incubating volume. (**D**) The activity of AChE released by PIPLC and erythrocytes (RBC) membrane extraction. Error bars represent the standard deviation from three independent experiments.

Most of the AChE in the blood is located on the surface of erythrocytes and is mainly bound to the extracellular membrane through the phosphatidylinositol. Therefore, a certain pretreatment to dissociate it from the membrane is needed. Phosphatidylinositol can be degraded by PIPLC, so that AChE can be dissociated from the erythrocyte membrane [37]. However, the optimal incubation conditions of PIPLC need to be explored. Based on previous report, PIPLC needed to be incubated with erythrocytes at 37 °C to release AChE. Erythrocytes needed to be washed with saline to remove the interfering substances in the plasma, but the frequency of washing cannot be excessive as it may lead to erythroclasis and hemolysis. In this study, the best incubation condition was determined by changing the concentration, time, and volume ratio of PIPLC and erythrocytes. As shown in Figure 4, the final concentration of PIPLC co-incubated with erythrocytes was 1 μg/mL (Figure 4A), the incubation time was 10 min (Figure 4B), and the volume ratio was 3:1 (Figure 4C). Compared with the method of extracting the erythrocyte membrane protein, the AChE activity of PIPLC method was 84.82% (Figure 4D). This suggests that the PIPLC method is feasible and highly efficient, making it suitable for subsequent experiments. For clinically diagnostic or monitoring purposes, blood samples obtained from patients can be processed using this procedure, enabling efficient and reliable analysis of AChE levels.

### 3.7. Precision, Quantitative Limits, and Linear Range

In this study, the intra-day and inter-day differences in the method were measured by low (400 ng/mL, 60 mU/mL), medium (800 ng/mL, 320 mU/mL), and high (2000 ng/mL, 800 mU/mL) concentrations of the AChE standard. As shown in Table 1 and Table 2, both relative error (RE) and relative standard deviation (RSD) values did not exceed 15%, indicating that the method is accurate, precise, and meets the requirements of sample determination.

The minimum quantitative limits of this method were 263.37 ng/mL and 39.02 mU/mL, respectively, and both the RE and RSD values remain below 20%, confirming the preservation of accuracy and repeatability, even at these lowermost quantifiable thresholds. The established determination range spans from 263.37 ng/mL to 3000 ng/mL for concentration, and from 39.02 mU/mL to 1000 mU/mL for activity, this ensured the generation of trustworthy and consistently reproducible confirmatory analysis outcomes within these intervals. Although the sensitivity for AChE activity was not as high as other newly developed methods, our method has provided sufficient sensitivity for blood AChE evaluation. Uniquely, this approach facilitates the prompt and concurrent assessment of AChE activity and concentration, yielding stable, dependable, and reproducible results for confirmatory analyses.

### 3.8. The Correlation Between SPR, ELISA, and Ellman’s Assay in AChE Detection

**Figure 5:**
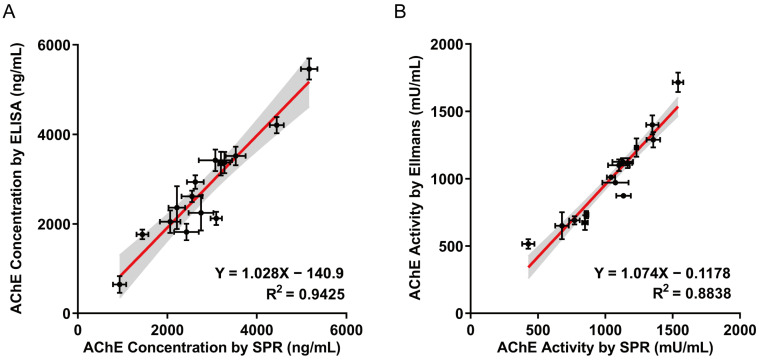
Comparison of methods for blood sample analysis. (**A**) Correlation between SPR and ELISA in detecting AChE concentration in human blood samples. (**B**) Correlation between SPR and Ellman’s assay in detecting AChE activity in human blood samples.

ELISA and Ellman’s assay are widely utilized methods for protein concentrations and enzymatic activities detecting, respectively. In this study, they are used as benchmarks to validate the accuracy and reliability of SPR technology. We evaluated the correlation between SPR and ELISA in detecting AChE concentrations in human blood samples (Figure 5A). Additionally, we investigated the correlation between SPR and the Ellman’s assay for detecting AChE activity in human blood samples (Figure 5B). The coefficient of determination (R^2^) between SPR and ELISA for detecting AChE concentrations and between SPR and Ellman’s assay for detecting AChE activity were 0.9425 and 0.8838, respectively (*p* < 0.0001). The results showed a strong linear correlation, indicating that our method can reliably measure AChE concentrations and enzymatic activities in human blood. The error bars visually demonstrate the differences in measurement errors between the two methods for the same sample. While ELISA and Ellman’s assay are reliable and widely used, they often involve complex sample preparation steps, lengthy incubation times, and reliance on specific reagents, which may introduce variability between experiments. In contrast, SPR offers significant advantages in terms of speed detection, higher precision, and repeatability, making it a valuable tool for rapid screening and high-throughput analysis.

### 3.9. Effects of AChE Inhibitors on AChE Activity and Content in Human Blood

**Figure 6:**
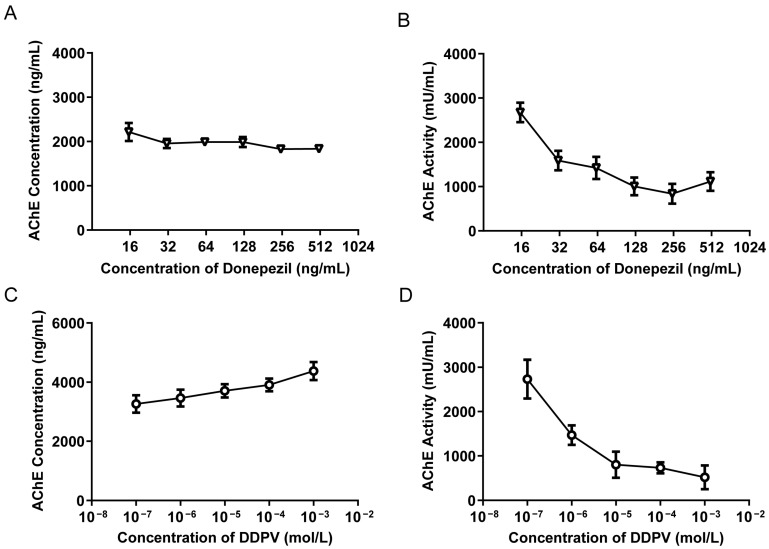
SPR detection of AChE activity and content in human blood after donepezil and dichlorvos treatment. (**A**,**C**) The blood AChE concentrations determined in different individuals. (**B**,**D**) The blood AChE activities determined in different individuals. Error bars represent the standard deviation from three or more independent experiments.

We applied the established method to study the effects of AChE inhibitors, donepezil, and dichlorvos (DDVP) on AChE in blood. By pre-treating human blood with different concentrations of donepezil or DDVP, the concentration and activity of AChE in the blood were measured. The results showed that as the concentration of donepezil or DDVP increased, the activity of AChE significantly decreased (Figure 6B,D). Notably, the reduction in AChE activity was much more pronounced with DDVP compared to donepezil. This could be attributed to the fact that DDVP is an organophosphorus pesticide that inhibits AChE activity by binding to the enzyme’s active site and forming a stable complex. This inhibition is irreversible, as it is difficult to dissociate DDVP from the enzyme once they are bound together [38,39]. In contrast, donepezil acts as a reversible inhibitor, allowing for the partial recovery of enzyme activity, even at higher concentrations. Interestingly, there was no significant change in AChE content after pre-treatment with donepezil (Figure 6A), but an increase in the concentration of DDVP led to an increase in AChE content (Figure 6C). This increase in AChE concentration after DDVP treatment may be attributed to conformational changes in the enzyme induced by the irreversible binding of DDVP to its active site. Such changes could expose previously hidden epitopes, enhancing the binding affinity of the detection antibody and leading to an overestimation of AChE levels. This interpretation is consistent with prior studies suggesting that structural alterations in enzymes can significantly affect antibody recognition [40]. Further structural and biochemical studies are needed to confirm this hypothesis and fully elucidate the mechanism. These findings underscore the distinct inhibitory mechanisms of donepezil and DDVP, as well as the sensitivity of our method in capturing these differences in AChE activity. These findings highlight the sensitivity of our detection method in identifying variations in AChE expression and activity. The distinct inhibitory mechanisms of donepezil and DDVP underscore the potential utility of this approach in evaluating AChE inhibitors and their effects in both research and clinical applications.

The methodology we utilized offers convenience, speed, and precision, making it ideal for rapid detection applications. It can be used to analyze the inhibitory effects of AChEIs on AChE activity in vivo, suitable for the analysis of a large number of samples in clinical trials, and also beneficial to the development of AChEIs. Additionally, it can be used to detect blood samples from AD patients who have been using AChEIs for a long time, helping to assess treatment efficacy and the early detection of drug side effects, allowing timely interventions. In the future, it is also expected to be used for the rapid detection of AChE in patients with organophosphorus pesticide poisoning.

## 4. Conclusions

In this study, a new method for the detection of AChE content and activity was established by the combination of SPR and the fluorescence detector. First, the anti-AChE antibody, immobilized to the CM5 sensor chip, could specifically recognize the AChE, and thus capture it on the chip and obtain the content of AChE in the blood. Then, the substrate ATCI were injected into the system and hydrolyzed by AChE. The product, the recovered thiocholine, reacted with a chromogenic agent. The activity of AChE can be determined according to the fluorescence intensity. This method provides convenient, rapid, and accurate approach for detecting AChEIs inhibitory activity in vivo. Moreover, by enabling the early warning and monitoring of adverse reactions, it plays a crucial role in the development and clinical application of AChEIs. Furthermore, as an SPR-based fluorescence detection method, it can be applied to other key neurotransmitter-related enzymes, broadening its potential applications in neuropharmacology.

## Figures and Tables

**Table 1:** Intra-day and inter-day precision and accuracy of the concentration of AChE.

Concentration (ng/mL)	Intra-Day (%)		Inter-Day (%)	
	RE	RSD	RE	RSD
400	−13.42	6.65	−13.92	11.14
800	5.79	6.20	3.07	3.22
2000	2.72	1.79	1.82	0.96

RE: relative error; RSD: relative standard deviation.

**Table 2:** Intra-day and inter-day precision and accuracy of the activity of AChE.

Activity (mU/mL)	Intra-Day (%)		Inter-Day (%)	
	RE	RSD	RE	RSD
60	−10.32	11.38	−14.46	13.41
320	3.74	5.51	4.48	9.65
800	−0.72	4.58	−3.81	9.21

RE: relative error; RSD: relative standard deviation.

## Data Availability

The data are available on reasonable request from the corresponding author.

## Supplementary Materials

The following supporting information can be downloaded at: https://www.mdpi.com/article/10.3390/bios15020118/s1, Figure S1: The coupling of anti-AChE antibody on CM5 chip; Figure S2: The binding of AChE to anti-AChE antibody immobilized on CM5 chip; Figure S3: The binding of different samples to anti-AChE antibody MAB303 immobilized on CM5 chip.
