# Supplementary material for: Dual Monitoring of Blood Acetylcholinesterase Content and Catalytic Activity Utilizing Fluorometry-Integrated Surface Plasmon Resonance
Source: Biosensors (Basel). 2025 Feb 17;15(2):118. doi: 10.3390/bios15020118 (PMC11853072; doi:10.3390/bios15020118)
Supplement: Supplementary file 1 [file biosensors-15-00118-s001.zip › biosensors-3406786-supplementary.pdf]

Dual Monitoring of Blood Acetylcholinesterase Content and Catalytic Activity Utilizing Fluorometry-Integrated Surface Plasmon Resonance

Yuanyuan Xie [1,†], Yifei Hou [2,†], Mengwei Hu [3,4,†], Hongzhuan Chen [1,5,6], Hao Wang [3], Lanxue Zhao [3,*] and Jianrong Xu [1,5,6,*]

[1] School of Integrative Medicine, Shanghai University of Traditional Chinese Medicine, Shanghai 201203, China
[2] Institute of Interdisciplinary Integrative Medicine Research, Shanghai University of Traditional Chinese Medicine, Shanghai 201203, China
[3] Department of Pharmacology and Chemical Biology, Shanghai Jiao Tong University School of Medicine Shanghai 200025, China
[4] Clinical Trial Center of Zhongnan Hospital, Wuhan University, Wuhan 430071, China
[5] Shuguang Lab of Future Health, Shuguang Hospital, Shanghai University of Traditional Chinese Medicine, Shanghai 201203, China
[6] Shanghai Frontiers Science Center of TCM Chemical Biology, Shanghai University of Traditional Chinese Medicine, Shanghai 201203, China
* Correspondence: annzoor@sjtu.edu.cn (L.Z.); jianrong.xu@shutcm.edu.cn (J.X.); Tel.: +86-18817302489 (L.Z.); +86-18621686069 (J.X.)
† These authors have contributed equally to this work and share first authorship.

Figure S1. The coupling of anti-AChE antibody on CM5 chip.

Figure S2. The binding of AChE to anti-AChE antibody immobilized on CM5 chip.

Figure S3. The binding of different samples to anti-AChE antibody MAB303 immobilized on CM5 chip.

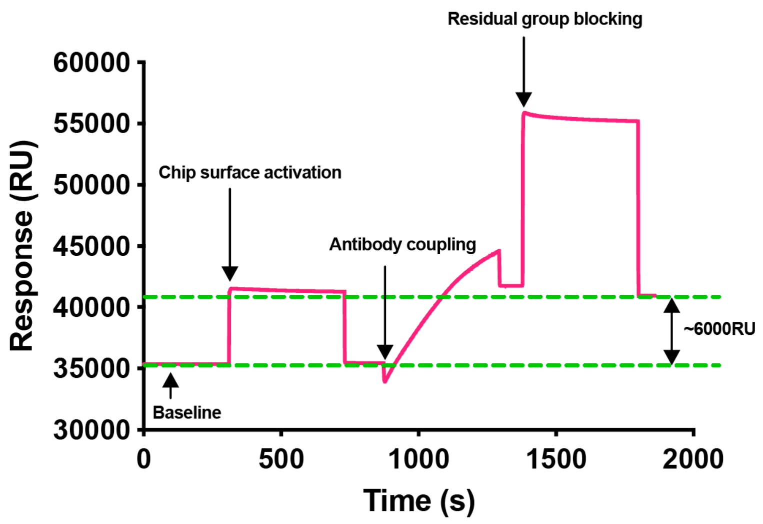

Figure S1. The coupling of anti-AChE antibody on CM5 chip.

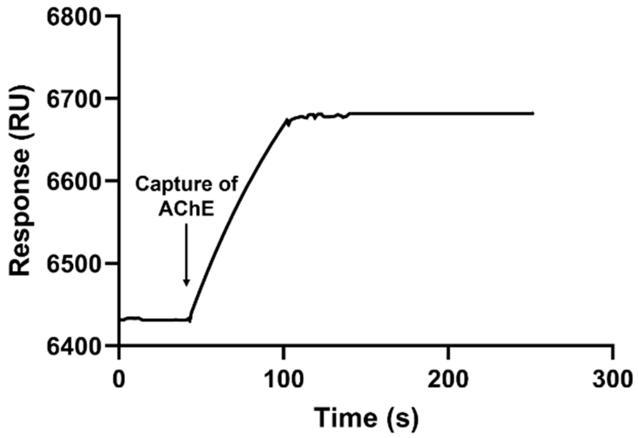

Figure S2. The binding of AChE to anti-AChE antibody immobilized on CM5 chip.

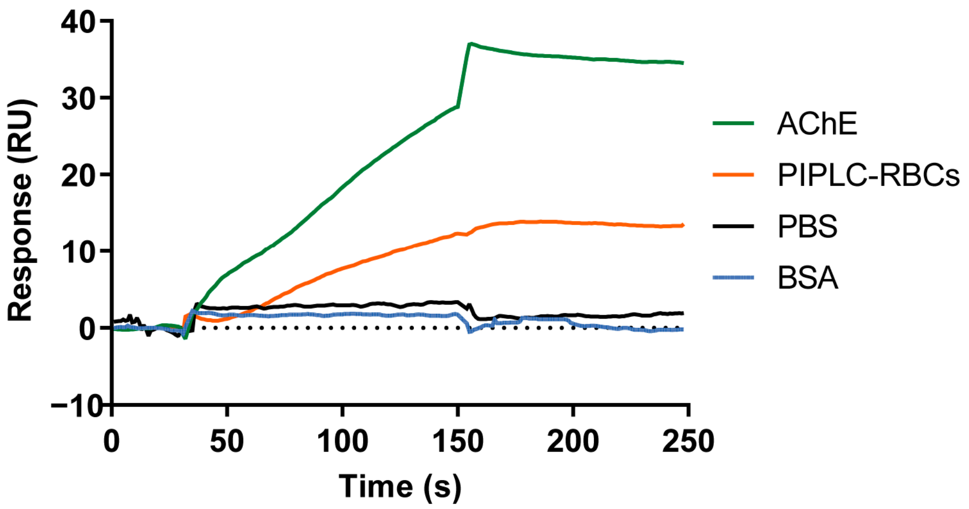

Figure S3. The binding of different samples to anti-AChE antibody MAB303 immobilized on CM5 chip.
